# Incorporating statistical strategy into image analysis to estimate effects of steam and allyl isocyanate on weed control

**DOI:** 10.1371/journal.pone.0222695

**Published:** 2019-09-30

**Authors:** Dong Sub Kim, Steven B. Kim, Steven A. Fennimore

**Affiliations:** 1 Department of Plant Sciences, University of California Davis, Salinas, California, United States of America; 2 Mathematics and Statistics Department, California State University, Monterey Bay, Seaside, California, United States of America; University of Wyoming, UNITED STATES

## Abstract

Weeds are the major limitation to efficient crop production, and effective weed management is necessary to prevent yield losses due to crop-weed competition. Assessments of the relative efficacy of weed control treatments by traditional counting methods is labor intensive and expensive. More efficient methods are needed for weed control assessments. There is extensive literature on advanced techniques of image analysis for weed recognition, identification, classification, and leaf area, but there is limited information on statistical methods for hypothesis testing when data are obtained by image analysis (RGB decimal code). A traditional multiple comparison test, such as the Dunnett-Tukey-Kramer (DTK) test, is not an optimal statistical strategy for the image analysis because it does not fully utilize information contained in RGB decimal code. In this article, a bootstrap method and a Poisson model are considered to incorporate RGB decimal codes and pixels for comparing multiple treatments on weed control. These statistical methods can also estimate interpretable parameters such as the relative proportion of weed coverage and weed densities. The simulation studies showed that the bootstrap method and the Poisson model are more powerful than the DTK test for a fixed significance level. Using these statistical methods, three soil disinfestation treatments, steam, allyl-isothiocyanate (AITC), and control, were compared. Steam was found to be significantly more effective than AITC, a difference which could not be detected by the DTK test. Our study demonstrates that an appropriate statistical method can leverage statistical power even with a simple RGB index.

## Introduction

Weed control is very important for crop management. Weed density counts or weed control efficacy has been traditionally quantified by manual weed density counts, and it is time-consuming and labor-intensive process. To overcome these challenges, photographic data taken by a digital camera can be used for quantifying weed densities instead of manual assessments. In recent years, many researchers have used image analysis techniques in agricultural research. For instance, Peña et al. (2015) detected weed plants with visible-light and multispectral cameras to obtain highly accurate results of weed detection [1]. Mahlein (2016) introduced RGB, spectral, thermal, fluorescence, and 3D sensors to detect color difference in cell, leaf, plant, plot, field, and ecosystem conditions [2].

Soil fumigants are an important part of the weed control program for strawberries in California. However, regular use of Methyl bromide (MB), the widely used soil fumigant for soil disinfestation in strawberry, has been discontinued in many countries in 2005 due to its ozone-depleting effect [3]. Steam and allyl-isothiocyanate (AITC) are being evaluated as alternatives to MB. Both methods are effective on weed control and do not deplete the ozone layer [4] [5].

RGB imaging is a simple method for quantifying a combination of red (R), green (G), and blue (B) colors. Most people have access to digital camera sensors in mobile phones or tablet PCs, and there are abundant image analysis programs available to analyze RGB data for plant recognition, identification, classification, and leaf area. Despite the technological advances, RGB data are often difficult to interpret due to lack of appropriate statistical methods for comparison. Downie et al. (2015) noted that limitations for image analysis still exist in statistical processes [6].

Some researchers have applied statistical hypothesis testing to RGB data. Woebbecke et al. (1995) tested and compared various color indices using t-tests to distinguish between a green plant from a nonplant background [7]. Golzarian et al. (2012) used the non-parametric Kruskal-Wallis and Friedman tests, which are alternatives to parametric ANOVA tests, to compare various color indices [8]. They used 240 digital images of wheat, ryegrass, brome grass, and wild oat, and they found that the 2G-R-B index (known as the excessive green index or EGI) is computationally simple and effective when the contrast between the background and the target is high. Longchamps et al. (2012) used the non-parametric Friedman test to compare the weed coverage in three different areas of ground by randomly selecting 110 images per plot [9].

In this article, RGB data were obtained from non-treated (control), steam, and AITC treatments. According to the previous studies, a traditional multiple comparison test, the Dunnett-Tukey-Kramer (DTK) test, and two alternative statistical methods, a bootstrap method and the Poisson model, were evaluated via simulation studies to compare their statistical power. The Poisson model can be applied to the case when the number of green spots is very low, and it was indicated in the current study. The two alternative statistical methods were preferred because they were more sensitive (i.e., higher statistical power). After confirming their advantage in the simulation studies, these two alternative statistical methods were applied to provide statistical evidence for effect of steam and AITC on weed control.

## Materials and methods

### Treatment description and application

Steam and Dominus (99.8% AITC) were applied on June 15, 2018. Microplots for this experiment were located at the US Department of Agriculture research station in Salinas, California, and each microplot was 1 m^2^. Each of the four treatments, control, Dominus, steam, and steam + Dominus, was replicated three times in a randomized complete block design. Steam was applied at a pressure of 0.8 bar for 60 minutes at the center of each microplot with a diesel-powered steam generator (SF-20, Sioux Corp, Beresford, SD). The microplots were covered with insulation mats for 24 h. HOBO data loggers (Onset Computer, Bourne, MA) were inserted at 0.3, 0.5, and 0.7 m from the center of each microplot. The maximum soil temperatures at 0.3, 0.5, and 0.7 m from the center were 99.6, 99.7, and 99.5°C, respectively, and times above 65°C were 457.3, 416.0, and 278.0 minutes, respectively. The maximum soil temperatures at 0.3 and 0.7 m from the center were 30.8 and 29.8°C in the control microplots. Dominus (18.7 mL m^−2^) was also applied at the center of each microplot by syringe with stylet at a depth of 15 cm.

### Data collection

Thirty-one days after treatment (DAT), each microplot was photographed by a digital camera (EOS 70D DSLR, Cannon, Inc., Tokyo, Japan). Each picture was converted from a JPEG file to a GIF image file for image analysis by Adobe Photoshop CC 2017 (Adobe Systems, San Jose, CA, USA). Each GIF file was uploaded to the image analysis program available at http://mkwak.org/imgarea/ developed by Dr. Minseok Kwak. The result of image analysis was copied to a spreadsheet of Microsoft Excel to identify the RGB code.

### Estimation of green area

**Fig 1:**
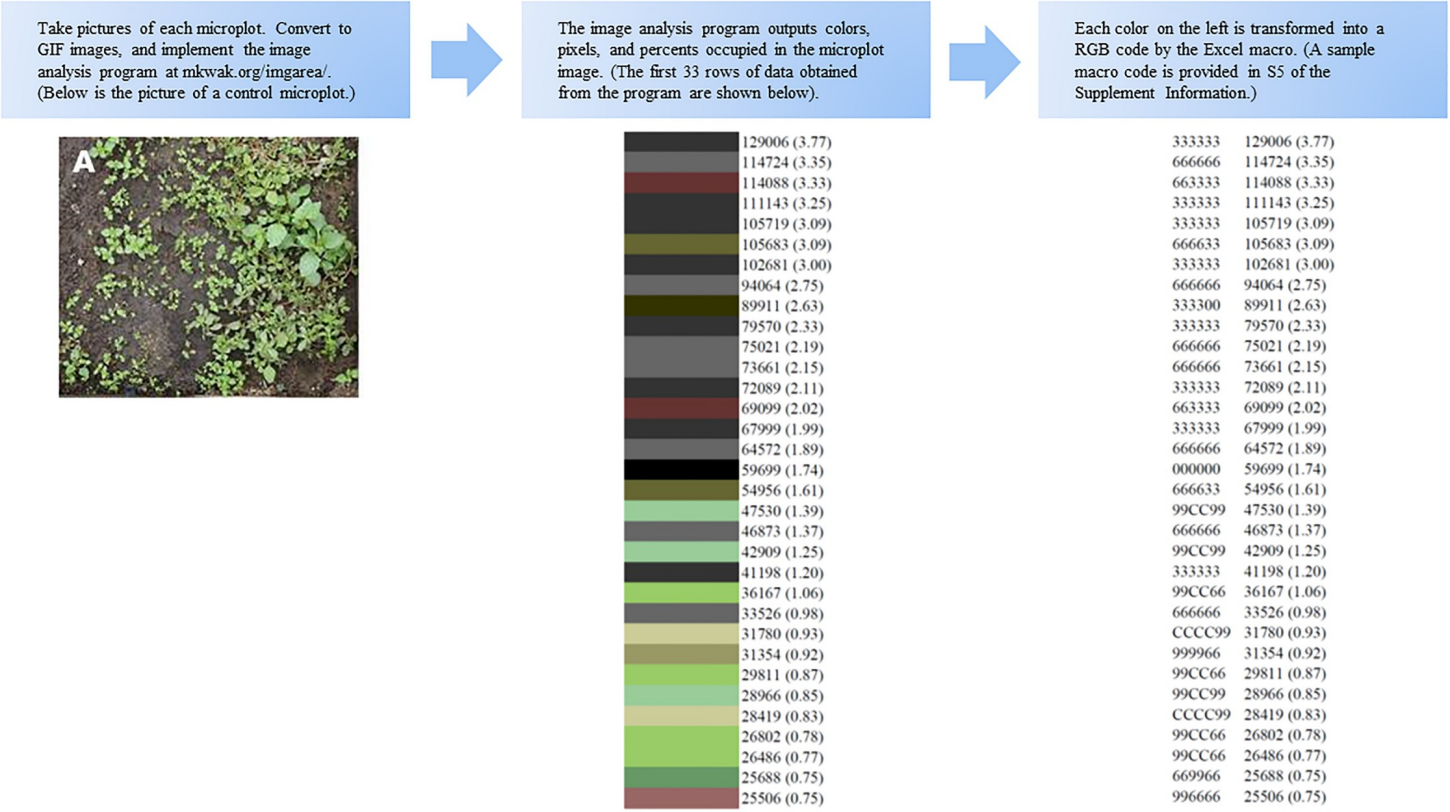
A schematic flowchart of the process. This schematic demonstrates the process of obtaining colors, pixels (percent occupied), and RBG codes.

**Fig 2:**
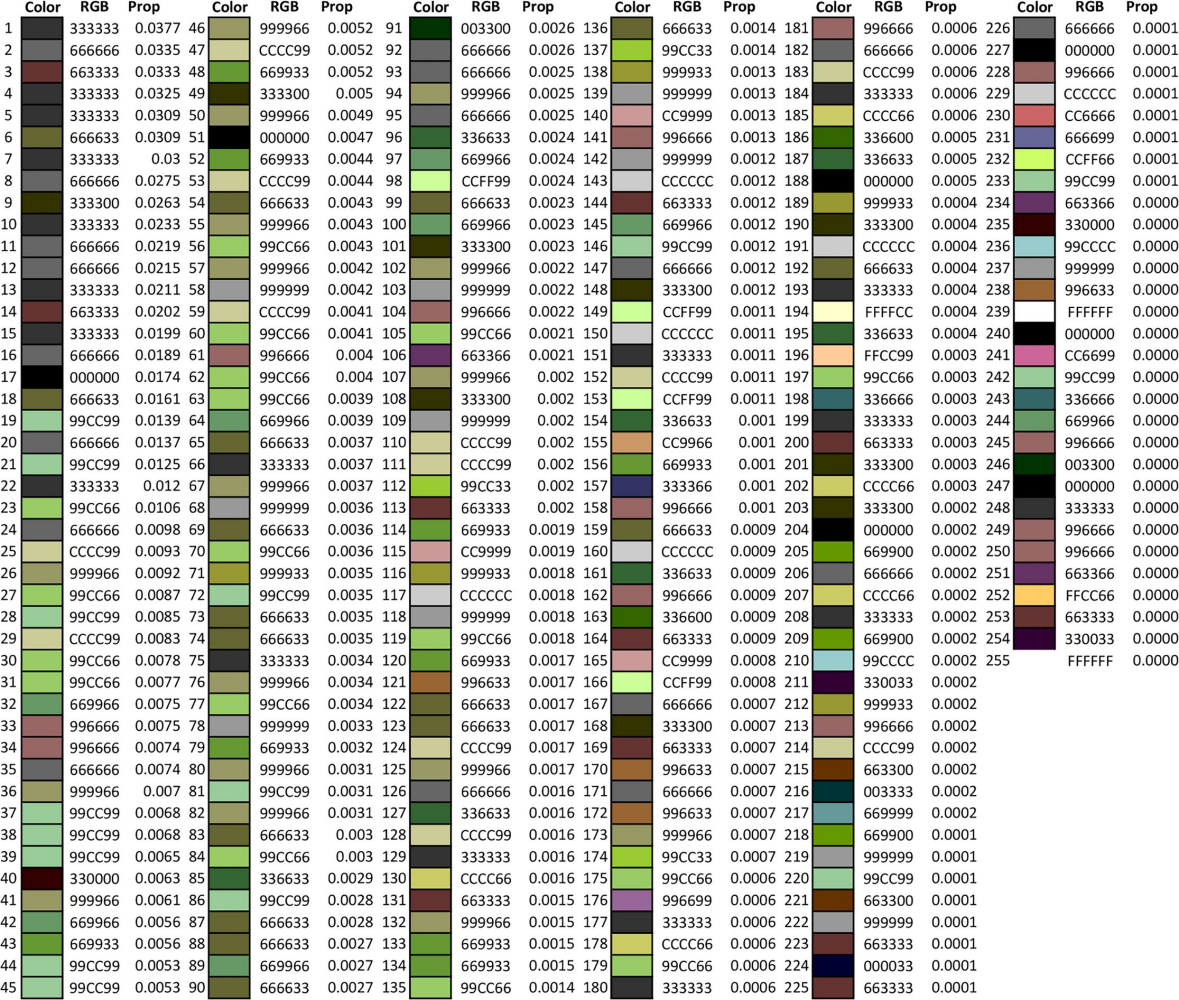
Sample RGB data. Each image was analyzed by calculating the proportion of 255 individual colors in each image (http://mkwak.org/imgarea/). This table is a result from image analysis of the picture shown in 1.

Fig 1 is a schematic flowchart diagram which demonstrates the process of obtaining colors, pixels, and RBG codes for a microplot by using the image analysis program (http://mkwak.org/imgarea/) and Excel Macro. Fig 2 provides full results (255 rows of data) obtained from the microplot shown in Fig 1. The image analysis program output 255 rows of data per microplot, and the Excel files in S1 and S2 Files include all data from each microplot. In Fig 2, the first color is close to black (RGB code of 333333), and the proportion occupied is 0.0377 (i.e., 3.37% of the observed microplot). The 42^nd^ color is close to green (RGB code: 669966), and the proportion occupied is 0.0056 (i.e., 0.56%). Among the various green colors observed, a common feature is a positive value of 2G – R – B which is known as an excess green index [7] [10] [11]. Defining a green color by G > R and G > B (i.e., both conditions to be met), the estimated proportion of green area is 0.2206 (22.06%).

### Challenge of comparative analysis

**Fig 3:**
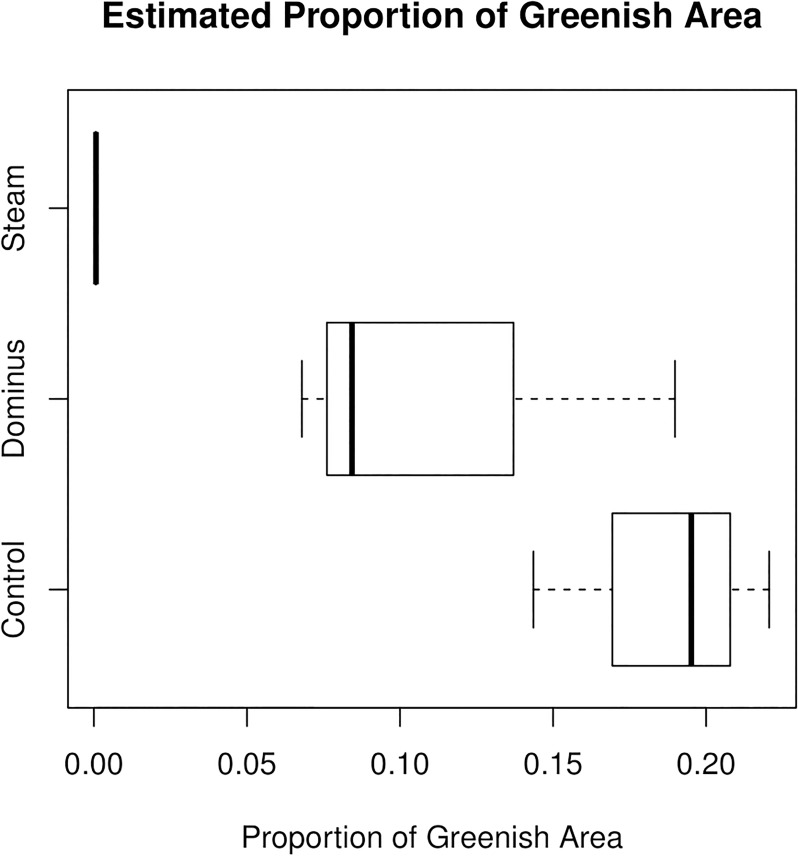
Estimated proportions of green area represented by green pixels. Boxplots for estimated proportions of green area (three microplots per group).

To formally test for differences among the three treatments (control, Dominus, and steam) on weed control, the proportion of green area, represented by the percent of pixels where *G* > *R* and *G* > *B*, were compared among the three treatments. From each microplot, the estimated proportion was 0.2206 (i.e., 22.06%), 0.1952, and 0.1436 in the control group (Group 0); 0.0843, 0.0679, and 0.1898 in the Dominus group (Group 1); and 0.0008, 0.0003, and 0.0007 in the steam group (Group 2). The nine data points, three per treatment, are shown in the left panel of Fig 3. In the Dominus group, the observed proportions of green area were slightly more variable than in the other two groups, and it could be due to random error within-microplot and between-microplot (e.g., Dominus low vapor pressure and limited movement in the soil). To test for statistical significance at *α* = 0.05, the Dunnett-Tukey-Kramer (DTK) test was used [12]. The DTK test is a popular hypothesis testing method which performs multiple pairwise comparisons with unequal variance assumption. As shown in the right panel of Fig 3, the difference in the two proportions was estimated as –0.072 with 95% CI (-0.334, 0.189) for comparing Group 1 to 0; –0.185 with 95% CI (–0.319, –0.052) for comparing Group 2 to Group 0; and –0.113 with 95% CI for (–0.338, 0.112). At *α* = 0.05, the difference between steam and control was statistically significant, but the other pairwise differences were not statistically significant. Though the effect of steam seems different from the effect of Dominus according to the boxplot, the statistical power was low due to a small number of microplots (three per group). The DTK test, which treated the entire data as a sample of size nine (three per group), did not utilize all information contained in the RGB data for the hypothesis testing. In agricultural experiments, increasing sample size is not always an option due to cost, space, time, and logistics. Given the small sample size, it is important to use statistical methods with enhanced power in order to overcome the practical challenges.

## Statistical methods

For the DTK test discussed in the previous section, an estimated proportion of green area (e.g., 0.2206) is treated as a single data point. In other words, the DTK test ignores individual pixel values for each green color obtained from the image analysis program, and it treats the entire data as a sample of size three per treatment. In this section, in order to utilize detail information contained in the RGB data, a bootstrap method and a Poisson model are considered for pairwise comparisons. The bootstrap method estimates and compares the proportion of green area, and a Poisson model estimates and compares the expected count of a small green spot in the case of a rare occurrence of weed. In a later section, the operating characteristics of the bootstrap method and the Poisson model (e.g., Type I error rate and statistical power) are compared to the operating characteristics of the DTK test by simulation studies.

The non-numeric values (RGB codes) have been transformed to the proportion of green area which is a numeric value between zero and one. The general structure of the data per treatment group is demonstrated in Table 1. In the table, let *R*_*i*_, *G*_*i*_, and *B*_*i*_ denote the decimal code of red, green, and blue in the *i*^th^ row. Let *W*_*i*_ denote the weight of the *i*^th^ row such that $\sum_{i=1}^{n}W_{i}=1$. For example, in a previous section, *W*_42_ = 19151/3422208 = 0.0056 was calculated. According to the RGB decimal code, *Z*_*i*_ = 1 if *G*_*i*_ > *R*_*i*_ and *G*_*i*_ > *B*_*i*_ and *Z*_*i*_ = 0 otherwise. Then the proportion of green area in the microplot is estimated by the weighted sum $S=\sum_{i=1}^{n}W_{i}Z_{i}$.

**Table 1:** Data structure in a spreadsheet.

Row	Red	Green	Blue	Weight	Binary
1	*R*_1_	*G*_1_	*B*_1_	*W*_1_	*Z*_1_
2	*R*_2_	*G*_2_	*B*_2_	*W*_2_	*Z*_2_
⋮	⋮	⋮	⋮	⋮	⋮
*n*	*R*_*n*_	*G*_*n*_	*B*_*n*_	*W*_*n*_	*Z*_*n*_

### Bootstrap confidence intervals

Let *H*_0_ denote the null hypothesis, the proportion of green area is the same across all groups (i.e., no effect of steam and Dominus on weed control). Let *H*_1_ denote the alternative hypothesis. Let *θ*_0_, *θ*_1_, and *θ*_2_ denote the proportion of green area in Group 0, Group 1, and Group 2, respectively. For the bootstrap method, the null hypothesis is *H*_0_: *θ*_0_ = *θ*_1_ = *θ*_2_, and the alternative hypothesis is *H*_1_: *θ*_0_ ≠ *θ*_1_, *θ*_0_ ≠ *θ*_2_, or *θ*_1_ ≠ *θ*_2_. To construct a confidence interval (CI) for *θ*_0_, *θ*_1_, *θ*_2_, or any parameter in terms of *θ*_0_, *θ*_1_, and *θ*_2_, a bootstrap method is considered [13] [14]. A bootstrap sample refers to a sample of size *n* drawn from the original sample of size *n* with replacement. Let $W_{i}^{*}$ and $Z_{i}^{*}$ denote the *i*^th^ weight and the binary outcome, respectively, in a bootstrap sample for *i* = 1,…, *n*. For a bootstrap sample, $\sum_{i=1}^{n}W_{i}^{*}=1$ is not guaranteed, and each $W_{i}^{*}$ can be normalized such that $\sum_{i=1}^{n}W_{i}^{*}=1$ in a bootstrap sample. Then $S^{*}=\sum_{i=1}^{n}W_{i}^{*}Z_{i}^{*}$ can be calculated from a bootstrap sample. By generating bootstrap samples a large number of times, CIs can be constructed for *θ*_0_, *θ*_1_, *θ*_2_, and any combination (e.g., *θ*_1_/*θ*_0_).

There are several kinds of bootstrap CI, and the method of bias-corrected and accelerated (BCa) bootstrap CI is considered [15]. It is known to be a robust method particularly when the sampling distribution is unknown or difficult to be represented mathematically [16]. Since the sampling distribution of *S* is not easily characterized, the BCa method would be an appropriate bootstrap method in our case. The computation can be done by the “boot” package in R version 3.3.3 [17] [18]. As a side note, other estimators for *θ*_*j*_ (*j* = 0, 1, 2) could be considered than the weighted sum *S*. For instance, a microplot image could be divided up into multiple sections, calculate *S* for each section, then average *S*’s. Since the bootstrap method is fairly robust, the bootstrap method could be utilized with an alternative estimator for *θ*_*j*_.

For comparing three groups, three pairwise comparisons are needed, namely *θ*_1_ versus *θ*_0_, *θ*_2_ versus *θ*_0_, and *θ*_2_ versus *θ*_1_. For constructing 95% CIs for the multiple comparisons, the individual confidence level can be adjusted at 1 − *α*/*m* = 0.983 by using the Bonferroni’s correction. In other words, a 98.3% CI is constructed for each pairwise comparison, and *H*_1_ is concluded if at least one of three CIs excludes a null value.

The null hypothesis can be formulated as *H*_0_: *μ*_*jk*_ = 1 for all *j* ≠ *k*, where *μ*_*jk*_ = *θ*_*j*_/*θ*_*k*_, the relative proportion of green area when group *j* is compared to group *k*. The parameter *μ*_*jk*_ can be estimated by *S*_*j*_/*S*_*k*_, where *S*_*j*_ and *S*_*k*_ are estimated proportions of green area in groups *j* and *k*, respectively.

The method of statistical hypothesis testing described in this section can be summarized as (1) obtaining bootstrap distributions of *S*_0_, *S*_1_, and *S*_2_, (2) calculating BCa bootstrap CIs for *μ*_10_, *μ*_20_, and *μ*_21_ with the Bonferroni’s correction (“boot” package in R), and (3) drawing a conclusion based on resulting CIs. This method can be generalized for comparing two or more groups.

### Poisson model for rare green spots

When the proportion of green area is very low (i.e., rare weed spots), the bootstrap method may not be appropriate because most bootstrap samples will estimate a zero proportion. For example, if *H*_0_ is true with *θ*_0_ = *θ*_1_ = *θ*_2_ = 0.01, a point mass at zero will be observed in the distribution of *S*_*j*_. If all *θ*_*j*_’s are close to zero, a Poisson model can be an alternative approach. Instead of comparing the proportion of green area, the expected count of green spots can be compared. Under the Poisson model, *θ*_*j*_ is interpreted as the expected count per given experimental area. For hypothesis testing, the likelihood ratio test (LRT) is considered with the “glm” function in R. The LRT calculates the log-likelihood value under the null hypothesis *H*_0_, denoted by *l*_0_, and the log-likelihood value under the alternative hypothesis *H*_1_, denoted by *l*_1_. The LRT compares 2(*l*_1_ − *l*_0_) to the chi-square distribution with *m* − 1 degrees of freedom to calculate the p-value, where *m* is the number of groups (e.g., *m* = 3 in our case). If the p-value is less than *α* = 0.05, *H*_0_ is rejected.

## Simulations

In this section, three hypothesis testing methods, the bootstrap method, the Poisson model (with LRT), and the DTK test, are evaluated. Various scenarios were considered under *H*_0_ and under *H*_1_. In a case of *H*_0_, the probability of rejecting *H*_0_ (type I error rate) should be close to *α* = 0.05. In a case of *H*_1_, the probability of rejecting *H*_0_ (statistical power) should be high. The Bonferroni’s correction was used with the bootstrap method, and it was not for the Poisson approach because *H*_1_ of the LRT negates *H*_0_: *θ*_0_ = *θ*_1_ = *θ*_2_ (i.e., at least one pairwise difference is nonzero).

### Simulation designs

For simulations, 23 scenarios were considered, 6 scenarios under *H*_0_ and 17 scenarios under *H*_1_. In each treatment group, *n* binary variables (*Z*_*i*_ = 1 or *Z*_*i*_ = 0) were simulated with some true values of *θ*_0_, *θ*_1_, and *θ*_2_ for groups 0, 1, and 2, respectively. See the left columns of Tables 2 and 3 for the true values of *θ*_0_, *θ*_1_, and *θ*_2_ in each scenario. Here *Z*_*i*_ = 1 represents a spot with a green color, and *n* weights were simulated by a beta distribution. To create a scenario close to the observed data, a beta distribution was fitted to the actual data (parameters were estimated by the methods of moment), *n* weights were generated from the beta distribution, then the weights were normalized such that $\sum_{i=1}^{n}W_{i}=1$. The sample size was fixed at *n* = 765 per group as in our actual data (255 data points per microplot × 3 microplots). Each scenario was repeated 1,000 times to estimate the probability of rejecting *H*_0_ in each scenario. Two thousand bootstrap samples were used to construct bootstrap CIs.

**Table 2:** Probability of rejecting *H*_0_ when *H*_0_ is true (type I error rate).

Scenario	*θ*_0_	*θ*_1_	*θ*_2_	Bootstrap	Poisson	DTK
1	0.500	0.500	0.500	0.047	0.002	0.004
2	0.250	0.250	0.250	0.053	0.028	0.007
3	0.100	0.100	0.100	0.043	0.042	0.011
4	0.050	0.050	0.050	0.049	0.039	0.010
5	0.025	0.025	0.025	0.069	0.049	0.012
6	0.010	0.010	0.010	NA*	0.052	NA*

* Not available due to numerical errors.

**Table 3:** Probability of rejecting *H*_0_ when *H*_1_ is true (statistical power).

Scenario	*θ*_0_	*θ*_1_	*θ*_2_	Bootstrap	Poisson	DTK
7	0.400	0.450	0.500	0.396	0.769	0.033
8	0.450	0.500	0.550	0.365	0.718	0.027
9	0.150	0.100	0.050	0.813	1.000	0.205
10	0.150	0.100	0.025	0.967	1.000	0.451
11	0.100	0.050	0.025	0.804	1.000	0.223
12	0.100	0.075	0.050	0.360	0.935	0.076
13	0.100	0.075	0.025	0.779	1.000	0.206
14	0.100	0.050	0.025	0.804	1.000	0.178
15	0.075	0.050	0.025	0.505	0.987	0.093
16	0.400	0.500	0.500	0.501	0.909	0.047
17	0.450	0.500	0.500	0.139	0.187	0.007
18	0.150	0.100	0.100	0.302	0.841	0.042
19	0.150	0.050	0.050	0.944	1.000	0.259
20	0.100	0.075	0.075	0.136	0.380	0.026
21	0.100	0.050	0.050	0.490	0.975	0.079
22	0.100	0.025	0.025	0.912	1.000	0.210
23	0.050	0.025	0.025	0.272	0.767	0.031

### Simulation results

The simulation results are reported in the right columns of Tables 2 and 3. Focusing on Scenarios 1 to 6 when *H*_0_ was true, the bootstrap method rejected *H*_0_ with a probability close to *α* = 0.05, except for Scenario 6. In Scenario 6 (*θ*_0_ = *θ*_1_ = *θ*_2_ = 0.01), the bootstrap method frequently resulted in a numerical error because *Z*_*i*_ = 1 occurred too rarely to calculate a bootstrap CI, and NA is reported in Table 2. Similarly, the DTK test could not be done for some simulated data because there was no variation in the estimated green proportions (i.e., all zeros), and NA is reported in the table as well. In such a case, the Poisson model resulted in the Type I error rate close to *α* = 0.05. The Poisson model showed lower type I error rates than *α* = 0.05 when *θ*_*j*_’s were relatively high. The DTK test consistently showed conservative results for all values of *θ*_*j*_’s.

In Scenarios 7 to 15 of the form *H*_1_: *θ*_*j*_ ≠ *θ*_*k*_ for all *j* ≠ *k*, the Poisson model consistently showed highest statistical power among the three hypothesis testing methods. The DTK test showed substantially lower statistical power than the bootstrap method and the Poisson model. In Scenarios 16 to 23 of the form *H*_1_: *θ*_1_ ≠ *θ*_2_ and *θ*_2_ = *θ*_3_, the Poisson approach still resulted in a higher statistical power than the bootstrap approach, and the DTK was not powerful. When Scenarios 13 and 20 are compared, resulting powers were generally lower in Scenario 20 because the values of *θ*’s were closer. Similar results were found when Scenarios 9 and 18 are compared and when Scenarios 14 and 21 are compared. See Table 3 for these results.

Regarding the Poisson approach giving a power equal to one in several settings but not in all settings, it would be reasonable to compare Scenarios 8 versus 9 (Table 3) and relate to the null scenarios (Table 2). As shown in Table 2, the Poisson approach resulted in a very low Type I error rate when *θ*’s were close to 0.5 (i.e., too conservative), and it resulted in a Type I error rate closer to the fixed significance level *α* = 0.05 as *θ*’s were closer to zero. The power in Scenario 8 (*θ*’s were close to 0.5) was substantially lower than the power in Scenario 9 (*θ*’s were closer to zero). In addition, although *θ*’s were evenly spaced by 0.05 in both Scenarios 8 and 9, the effect size was greater in Scenario 9 in terms of relative expected counts (i.e., a greater magnitude of *θ*_*j*_/*θ*_*k*_ in Scenario 9).

When simulations from Scenario 1 to 23 were replicated 10 times (1,000 times per replication per scenario), similar results were obtained. The replicated simulation results are included in the S5 File.

## Application

From the actual data described in a previous section, *μ*_01_, *μ*_02_, and *μ*_12_ were estimated as 1.57, 263.7, and 168.3, respectively, using the bootstrap method. When Group 0 (control) was compared to Group 1 (Dominus), the estimated proportion of green area was 1.6 times greater. When Group 0 was compared to Group 2 (steam), it was 264 times greater. When Group 1 was compared to Group 2, it was 168 times greater. The resulting bootstrap CIs were (1.13, 2.29), (137.6, 726.0), and (90.4, 451.2), respectively. At significance level *α* = 0.05, all three pairwise groups were significantly different. This conclusion is different than the conclusion from the aforementioned DTK test. In particular, steam is substantially more effective than both control and Dominus, and Dominus is slightly more effective compared to control. Using the Poisson model with LRT, the resulting p-value was nearly zero, and the respective CIs for *μ*_*jk*_ were (1.17, 1.97), (7.78, 29.18), and (5.07, 19.43) which are different than the CIs using the bootstrap method. Recall that *θ*_*j*_ is interpreted as the proportion of green area in the bootstrap method, and it is interpreted as the expected count of green spots in the Poisson model.

**Fig 4:**
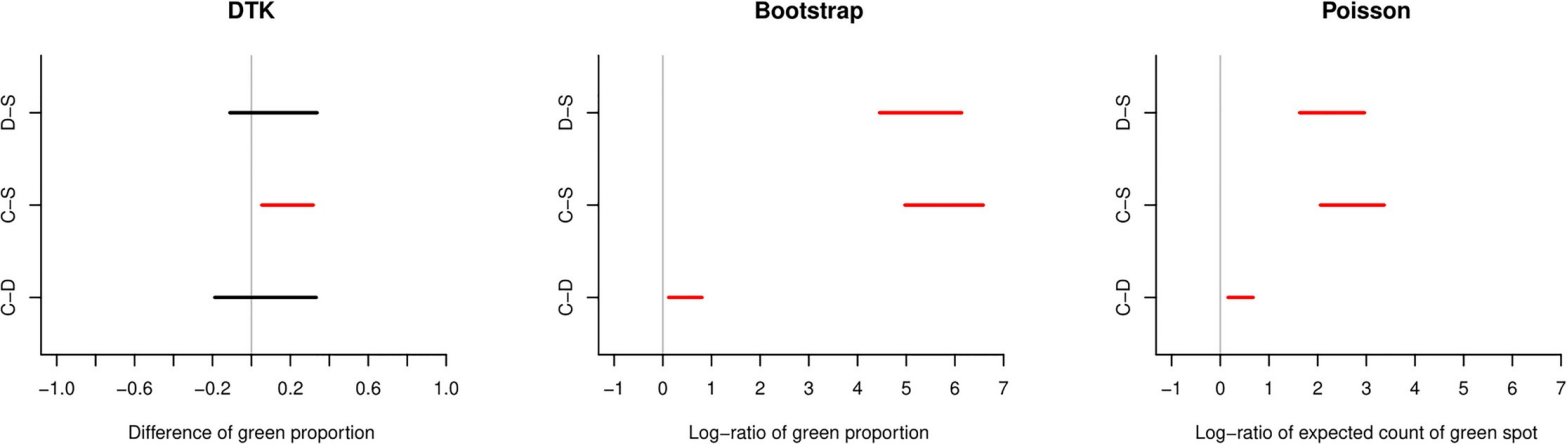
Resulting confidence intervals for the pairwise comparison. The DTK method (left) estimates the difference of green proportions, the bootstrap method (middle) estimates the ratio of green proportions (log-ratio in the figure), and the Poisson model (right) estimates the ratio of expected count of green spots. In the figure, C (control) is for Group 0, D (Dominus) is for Group 1, and S (steam) is for Group 2.

Fig 4 graphically presents the resulting CIs from the DTK method (left), the bootstrap method (middle), and the Poisson model (right). To avoid the extremely large magnitude of the CIs in the figure, the resulting CIs for *μ*_*jk*_ were log-transformed, so the null value became zero after the log-transformation in the bootstrap method and the Poisson model.

In the data collection, there was an additional experimental group, a combination of steam and Dominus (referred to as Group 3). In three microplots of Group 3 (steam + Dominus), the observed green areas were 0.0004, 0.0006, and 0.0004. In the three microplots of Group 2 (steam only), recall that the observed green areas were 0.0008, 0.0003, and 0.0007. Since these are very low proportions (i.e., *W*_*i*_ is close to zero for *Z*_*i*_ = 1), the Poisson model would be more appropriate to compare these two experimental groups. *μ*_23_ was estimated as 0.82 with a 95% CI (0.41, 1.67). As shown in S1 Fig, the effect of steam was nearly 100% effective by itself, so it would make sense that Dominus would not improve upon the effect of steam. It appears that the estimated *μ*_23_ is accurate under the Poisson model.

All supporting information including data and R code are available in S1 Fig, S1, S2 and S3 Files, and S1 Code.

## Discussion

Woebbecke et al. (1995) reported that the 2G-R-B index (known as the excessive green index or EGI) or the modified hue was sensitive for separation of plants and soil [7], so the similar idea was applied for separation of weeds and soil in this study. Although they used a modified version of the EGI, Golzarian et al. (2012) reported that the EGI is computationally simple and effective when the contrast between the target (weed) and the background (soil) is high [8]. Among various indices for defining “green,” the simple definition “G > R and G > B” was used in this study, and the effect of steam was statistically significant based on the bootstrap approach and the Poisson approach. These approaches also detected the small effect of Dominus based on three microplots per group. These results reflect that an appropriate statistical method can leverage the sensitivity of simple color indices. Our supplementary experiment showed that even smart phones are highly reliable for estimating the proportion of green area with an intraclass correlation of 0.9 when three randomly chosen smartphones were tested [19] [20] [21]. (The data of supplementary experiment are available in S4 File.) Therefore, the simple color indices, together with the statistical methods demonstrated in this article, can be an alternative strategy for examining the effects of weed control and other similar types of comparative analysis, particularly when a large sample size is not available. Most of the aforementioned studies focused on the technical advantages of various color indices with a less of an emphasis on statistical methodology to detect the effect of a treatment.

There are many statistical methods available for comparing two or more treatments such as ANOVA with Tukey’s range test, Dunnett-Tukey-Kramer (DTK) test, Kruskal-Wallis test, and Friedman test [22], [12] [23] [24] [25] [26]. If one of these statistical methods was considered, the RGB data would be treated as a sample of size three per treatment, and the hypothesis testing would suffer from low statistical power. For instance, the DTK test is a popular hypothesis testing method in agricultural studies to test for difference in means under the normality assumption with unequal variances [12]. With such a small sample size per group, it is difficult to check the normality assumption, and even if the assumption is true, there should be low statistical power for comparing treatment effects. To this end, two alternative statistical methods, bootstrap method and Poisson model, were considered for the RGB data. The simulation results demonstrated that the bootstrap method and the Poisson model provide higher statistical power while preserving the type I error rate at *α* = 0.05. In the applied example, the DTK only detected the difference between steam and control, but the bootstrap method and the Poisson model detected the difference among steam, Dominus, and control. In addition, unlike the non-parametric tests (e.g., Kruskal-Wallis and Friedman), the bootstrap method and the Poisson model can address the research objective with interpretable parameters. The bootstrap approach can estimate the relative proportion of weed area between two treatments, and the Poisson approach can estimate the relative average count of weed per given area between two treatments when the occurrence is rare. After determining the outperformance of these alternative methods when compared to the DTK test via the simulation studies, they were applied to the RGB data to compare the three treatment groups on weed control.

In the simulation study presented in this article, a number of assumptions were simplified, and more investigations are needed under weaker assumptions. In the weed control experiment presented in this article, all microplots were located close to each other (about one meter apart between two neighboring microplots), and they were completely randomized to one of the treatments. Because it was an outdoor experiment, there could be additional random error among the microplots due to environmental factors and other unknown factors. For instance, the weed species composition was not the same in all plots which may have contributed to variation. If these factors are significant, the Poisson model approach described in this manuscript can lead to an inflated Type I error rate in over-dispersed data. According to the simulation study, the Poisson approach outperforms the bootstrap approach in terms of both Type I error and power. In a case of severe over-dispersion, the Type I error rate should be controlled, and a quasi-Poisson model or a generalized linear mixed model may be considered alternatively. Comparing to these alternative approaches, the relative performance of the bootstrap approach remains unknown, and it is a potential topic for future research. In the simulation study, the Bonferroni’s correction was used for multiple comparisons, and an alternative correction method could be used. Benjamini and Hochberg (1995) discussed another procedure of multiple comparisons based on ranking p-values [27]. The choice of a correction method was not a main focus in this article, but it is an important topic in agricultural studies which involve many comparisons. The procedure of ranking p-values also controls the false discovery rate (i.e., Type I error rate), and it may be more powerful particularly when the number of comparisons is very large. Despite the fact that the estimated green proportions were generally lower in the Dominus group than in the control group, the observed variance was greater in the Dominus group. If it was the case for the count data (i.e., lower expected counts with higher variance), the data could not conform the mean-variance relationship of the Poisson model. In this case, the zero-inflated Negative-Binomial distribution would be a more flexible approach than the Poisson distribution, and this comparison was not made in the simulation study. Future research should focus on the impact of the simplified assumptions when the truth is more complex, and any negative consequences (e.g., an inflation of Type I error, a loss of statistical power) should be addressed by more sophisticated statistical methods than the methods discussed in this article.

## Conclusion

The bootstrap method and the Poisson model (particularly for rare green spots of weed occurrence) result in substantially higher statistical power than the DTK test. The bootstrap method and the Poisson model are more powerful than the DTK test because they utilize detail information (pixels) obtained from each microplot, whereas the DTK test treats an observed green proportion in a microplot as a single data point. Using the bootstrap method and the Poisson model, it was evident that weed control with steam is effective for at least 31 days, and weed control with steam is significantly more effective than weed control with Dominus. The powerful statistical methods described here are recommended particularly when the sample size is small. The combination of the RGB analysis and the statistical methods can be useful for various purposes such as interim evaluations (e.g., monitoring and quantifying color changes until a final harvest) and final evaluations in a large field (e.g., areas where it is practically impossible to count weeds).

## Supporting information

S1 Fig Pictures of microplots. Pictures taken by a digital camera in three microplots per treatment group (control, Dominus, steam, and steam + Dominus).
(TIF)

S1 File Colors. Colors exported by the Image Area Analyzer (http://mkwak.org/imgarea/) when the picture from each microplot was uploaded in the website.
(XLSX)

S2 File Pixels. Pixels exported by the Image Area Analyzer (http://mkwak.org/imgarea/) when the picture from each microplot was uploaded in the website.
(XLSX)

S3 File RGB codes. RGB codes created by Excel macro [28]. Each column of col.xlsx, pix.xlsx, and rgb.xlsx are from the same microplot.
(XLSX)

S4 File Data. Data to test the reliability of using smart phones (estimated green proportions in twenty plots by three smart phones).
(XLSX)

S5 File Replicated simulation results. Simulation results replicated 10 times.
(XLSX)

S1 Code Sample R code. R code used to produce results in the section of Application.
(R) Click here for additional data file.

## References

[1] Peña JM, Torres-Sȧnchez J, Serrano-Pėrez A, de Castro AI, Lȯpez-Granados F. Quantifying efficacy and limits of unmanned aerial vehicle (UAV) technology for weed seedling detection as affected by sensor resolution. Transactions of the ASAE. 2015;15(3):5609–5626. 10.3390/s150305609 PMC4435221 25756867

[2] Mahlein AK. Plant disease detection by imaging sensors-parallels and specific demands for precision agriculture and plant phenotyping. Plant Disease. 2016;100(2):241–251. 10.1094/PDIS-03-15-0340-FE 30694129

[3] Sen F, Meyvaci KB, Turanli F, Aksoy U. Effects of short-term controlled atmosphere treatment at elevated temperature on dried fig fruit.. Journal of Stored Products Research. 2010;46(1):28–33. 10.1016/j.jspr.2009.07.005

[4] Samtani JB, Ajwa HA, Weber JB, Browne GT, Klose S, Hunzie J, et al Evaluation of non-fumigant alternatives to methyl bromide for weed control and crop yield in California strawberries (Fragaria ananassa L.). Crop Protection. 2011;30(1):45–51. 10.1016/j.cropro.2010.08.023

[5] Bangarwa SK, Norsworthy JK, Gbur EE. Allyl isothiocyanate as a methyl bromide alternative for weed management in polyethylene-mulched tomato. Weed Technology. 2012;26(3):449–454. 10.1614/WT-D-11-00152.1

[6] Downie HF, Adu MO, Schmidt S, Otten W, Dupuy LX, White PJ, et al Challenges and opportunities for quantifying roots and rhizosphere interactions through imaging and image analysis. Plant, Cell and Environment. 2015;38(7):1213–1232. 10.1111/pce.12448 25211059

[7] Woebbecke DM, Meyer GE, Von Bargen K, Mortensen DA. Color indices for weed identification under various soil, residue, and lighting conditions. Transactions of the ASAE. 1995;38(1):259–270. 10.13031/2013.27838

[8] Golzarian MR, Lee MK, Desbiolles MA. Evaluation of color indices for improved segmentation of plant images. Transactions of the ASAE. 2012;55(1):261–273. 10.13031/2013.41236

[9] Longchamps L, Panneton B, Simard MJ, Leroux GD. Could weed sensing in corn interrows result in efficient weed control? Weed Technology. 2012;26(4):649–656.

[10] Meyer GE, Mehta T, Kocher MF, Mortensen DA, Samal A. Textural imaging and discriminant analysis for distinguishing weeds for spot spraying. Transactions of the ASAE. 1998;41(4):1189–1197. 10.13031/2013.17244

[11] Yang W, Wang S, Zhao X, Zhang J, Feng J. Greenness identification based on HSV decision tree. Information Processing in Agriculture. 2015;2:149–160. 10.1016/j.inpa.2015.07.003

[12] Dunnett CW. Pairwise multiple comparisons in the unequal variance case. Journal of the American Statistical Association. 1980;75(372):796–800. 10.1080/01621459.1980.10477552

[13] Efron B. Bootstrap methods: Another look at the jackknife. The Annals of Statistics. 1979;7(1):1–26. 10.1214/aos/1176344552

[14] Efron B, Tibshirani R. An introduction to the bootstrap. Boca Raton, FL: Chapman & Hall/CRC; 1993.

[15] Efron B. Better bootstrap confidence intervals. Journal of the American Statistical Association. 1987;82(397):171–185. 10.2307/2289153

[16] Haukoos JS, Lewis RJ. Advanced statistics: bootstrapping confidence intervals for statistics with “difficult” distributions. Academic Emergency Medicine. 2005;12(4):360–365. 10.1197/j.aem.2004.11.018 15805329

[17] Canty A, Ripley B. boot: Bootstrap R (S-Plus) functions. 2017; R package version 1.3-20.

[18] R Core Team. R: A language and environment for statistical computing R Foundation for Statistical Computing, Vienna, Austria 2017 URL https://www.R-project.org/.

[19] Ebel RL. Etimation of the reliability of ratings. Psychometrika. 1951;16:407–424. 10.1007/BF02288803

[20] Bartko JJ. The intraclass correlation coefficient as a measure of reliability. Psychological Reports. 1966;19:3–11. 10.2466/pr0.1966.19.1.3 5942109

[21] Koo TK, Li MY. A guideline of selecting and reporting intraclass correlation coefficients for reliability research. Journal of Chiropractic Medicine. 2016;15(2):155–163. 10.1016/j.jcm.2016.02.012 27330520 PMC4913118

[22] Tukey J. Comparing individual means in the analysis of variance. Biometrics. 1949;5(2):99–114. 10.2307/3001913 18151955

[23] Kruskal WH, Wallis WA. Use of ranks in one-criterion variance analysis. Journal of the American Statistical Association. 1952;47(260):583–621. 10.1080/01621459.1952.10483441

[24] Hollander M, Wolfe DA. Nonparametric statistical methods. New York: John Wiley & Sons; 1973.

[25] Kutner M, Nachtsheim C, Neter J, Li W. Applied linear statistical models (5th edition). New York, NY: McGraw-Hill/Irwin; 2004.

[26] Hecke TV. Power study of anova versus Kruskal-Wallis test. Journal of Statistics and Management Systems. 2012;15(2–3):241–247. 10.1080/09720510.2012.10701623

[27] Benjamini Y, Hochberg Y. Controlling the false discovery rate: a practical and powerful approach to multiple testing. Journal of the Royal Statistical Society, Series B. 1995;57(1):289–300.

[28] Wyatt A. Determining the RGB Value of a Color. Tips.Net. 19. Mar 2016. Available from: https://excelribbon.tips.net/T010180_Determining_the_RGB_Value_of_a_Color.html Cited 8 April 2019.

