# Supplementary figures and images for: Incorporating statistical strategy into image analysis to estimate effects of steam and allyl isocyanate on weed control
Source: PLoS One. 2019 Sep 30;14(9):e0222695. doi: 10.1371/journal.pone.0222695 (PMC6768448; doi:10.1371/journal.pone.0222695)

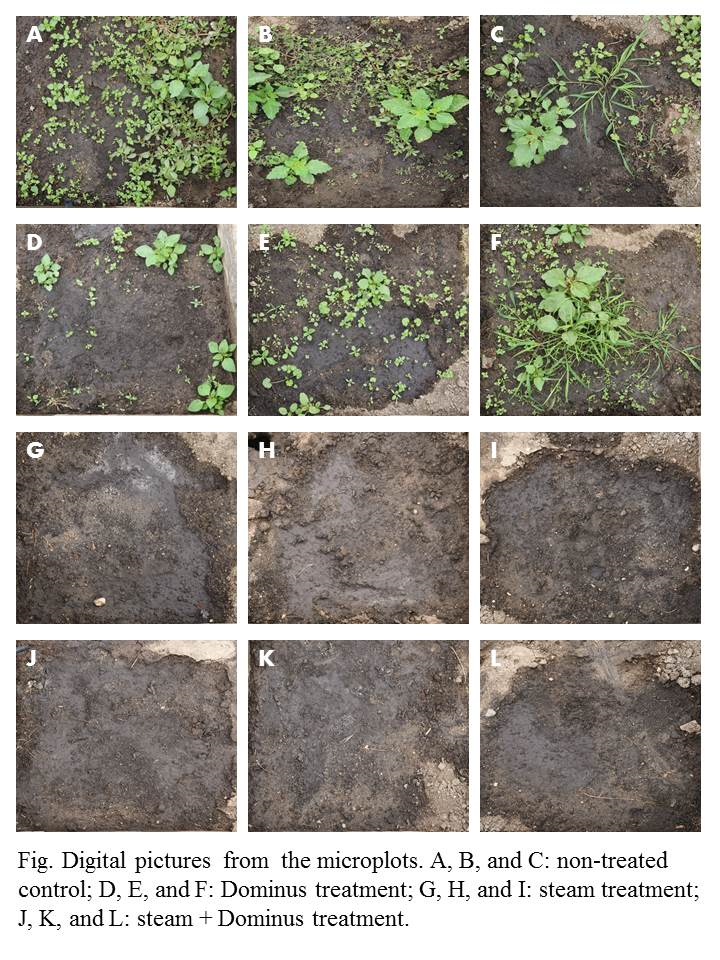

Supplement: S1 Fig — Pictures taken by a digital camera in three microplots per treatment group (control, Dominus, steam, and steam + Dominus). (TIF) [file pone.0222695.s001.tif]
